# Making sense of human colorectal cancer molecular subtypes: mice are stepping in

**DOI:** 10.1038/s41420-025-02594-7

**Published:** 2025-07-01

**Authors:** Caleb Green, Pamela Roccia, Alessandro Rufini

**Affiliations:** 1 Leicester Cancer Research Centre, University of Leicester, Leicester, UK; 2 Dipartimento di Bioscienze, University of Milan, Milan, Italy

**Keywords:** Cancer metabolism, Cancer models

Colorectal cancer (CRC) is the third most diagnosed cancer worldwide, accounting for 9.2% of all cancer mortalities [1]. In 2015, the consensus molecular subtype of CRC (CMS) identified 4 different subgroups based on subtype-specific gene expression profiles identified by RNA sequencing analysis [2]. CMS1, the immune subtype, exhibits frequent mutations of the *BRAF* oncogene, high levels of microsatellite instability (MSI) and a prominent immune infiltrate. CMS2, the canonical subtype, displays chromosomal instability and hyperactivation of WNT and MYC pathways. CMS3 is enriched for mutations in the *KRAS* oncogene and dysregulation of metabolic pathways. CMS4, the mesenchymal subtype, displays a robust stromal signature and epithelial-to-mesenchymal transition, leading to invasiveness and poor prognosis [2].

A recent publication from Torang et al. [3] aimed to untangle the mechanisms underpinning the establishment of CMS, exploiting an outstanding array of 16 genetically engineered murine intestinal organoids carrying different combinations of colorectal driver mutations [4]. Firstly, they developed a useful mouse CMS classifier from bulk RNA-seq data grouping organoids into 3 major CMS: CMS2, CMS3 and CMS4 (Fig. 1). By doing so, the authors reconciled human CMS classification with pre-clinical mouse models and outlined a striking association between targeted cancer-associated mutations and CMS. Indeed, loss of the tumour suppressor gene *Apc* resulted in activation of the Wnt pathway and the establishment of CMS2 organoids. *Kras* mutant organoids were classified as CMS3, and finally, organoids carrying activation of the Notch pathway were identified as CMS4 (unless loss of *Apc* was concomitant, in which case they were CMS2). Critically, these findings indicate that the cancer mutational profile drives CRC subtype specification in a cell-autonomous fashion, even in the case of stromal-rich CMS4 cancers, as organoid cultures comprise only intestinal epithelial cells. Interestingly, no genetic combination originated CMS1-like organoids. This could be due to the lack of immune infiltrates in organoid culture, but also to the inability to trigger MSI. Interestingly, a mouse knock-out for the Dipeptidase-1 (*DPEP1*) gene was recently shown to develop invasive tumours with features of MSI CRC [5]. It is tempting to speculate that, in combination with mutations in oncogenes linked to MSI cancers (e.g., *BRAF*), such models could recapitulate CMS1 CRC (Fig. 1).

**Fig. 1:**
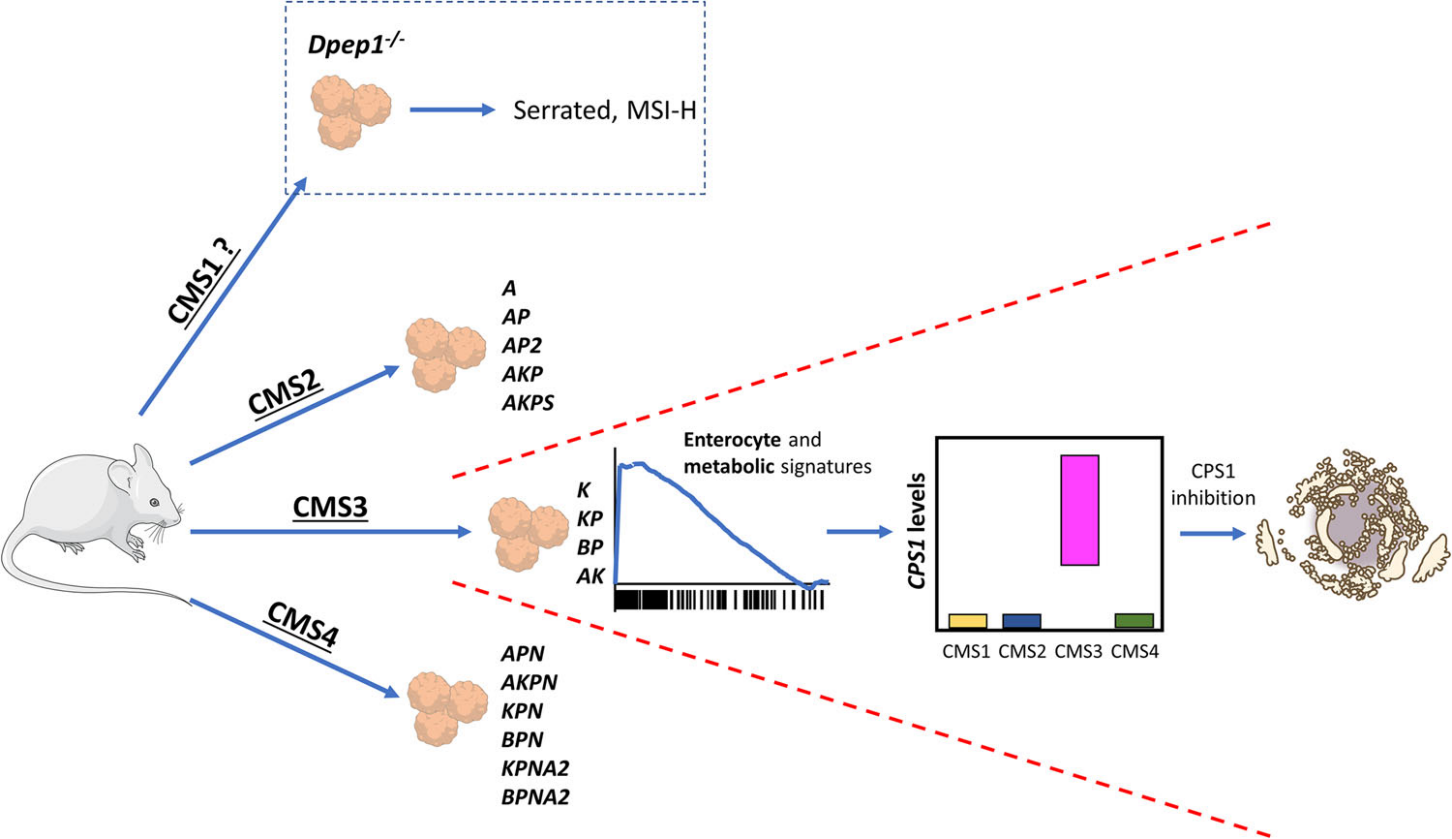
Genetically engineered mouse models recapitulate CRC subtypes. Organoids derived from mice harbouring cancer-related mutations recapitulate different molecular subtypes of CRC. Currently, no murine model reproduces the CMS1 subtype, although mice knockout for the *Dpep1* gene display microsatellite instability and represent a promising step forward towards the development of a mouse model of CMS1 CRC. Using transcriptomic profiles of organoids, Torang and colleagues identified enriched enterocyte and metabolic gene signatures in organoids of the CMS3 subtype, which led to the identification of increased expression of the *CPS1* gene in these cancers. Genetic and pharmacological inhibition of the CPS1 enzyme is potentially a novel therapeutic option for patients with CMS3 cancers. Illustration of apoptotic cells from NIAID NIH BIOART Source (bioart.niaid.nih.gov/bioart/71). We acknowledge Servier on Bioicons (www.bioicons.com) for the use of the mouse icon. A = *Apc*^*−/−*^, AP = *Apc*^*−/−*^; *Trp53*^*−/−*^, AP2 = *Apc*^*−/−*^; *Trp53*^*R172H/−*^, AKP = *Apc*^*−/−*^; *Kras*^*G12D/+*^; *Trp53*^*−/−*^, AKP2 = *Apc*^*−/−*^; *Kras*^*G12D/+*^; *Trp53*^*R172H/−*^, AKPS = *Apc*^*−/−*^; *Kras*^*G12D/+*^; *Trp53*^*−/−*^; *Smad4*^*-/+*^, K = *Kras*^*G12D/+*^, KP = *Kras*^*G12D/+*^;*Trp53*^*−/−*^, BP = *Braf*^*V600E/+*^; *Trp53*^*−/−*^, AK = *Apc*^*−/−*^; *Kras*^*G12D/+*^, APN = *Apc*^*−/−*^; *Trp53*^*−/−*^; *Notch1-ICD*, AKPN = *Apc*^*−/−*^; *Kras*^*G12D/+*^; *Trp53*^*−/−*^; *Notch1-ICD*, KPN = *Kras*^*G12D/+*^; *Trp53*^*−/−*^; *Notch1-ICD*, BPN = *Braf*^*V600E/+*^; *Trp53*^*−/−*^; *Notch1-ICD*, KPNA2 = *Kras*^*G12D/+*^; *Trp53*^*−/−*^; *Notch1-ICD*; *Alk5*^*−/−*^, BPNA2 = *BrafV*^*600E/+*^; *Trp53*^*−/−*^; *Notch1-ICD*; *Alk5*^*−/−*^.

Next, the authors focused on *Kras* mutant organoids of the CMS3 subtype. CMS3 is arguably the least characterised subtype and a clinical challenge, as patients suffering from this CRC subtype are unlikely to benefit from oxaliplatin-based adjuvant chemotherapy, the mainstay therapeutic treatment for CRC [6]. Torang and colleagues made two key observations. They confirmed the widespread rewiring of metabolic gene signatures in CMS3, and discovered that the metabolic pathways enriched in the CMS3 subtype are shared with normal enterocyte cells, steering the authors towards the surprising identification of increased expression of enterocyte gene signatures in CMS3 mouse organoids and human CRC (Fig. 1). The top-ranking metabolic gene expressed in CMS3 is carmaboyl-phosphate-synthase-1 (*CPS1*), a gene involved in the urea cycle and in pyrimidine biosynthesis. Interestingly, isotope tracing experiments showed that CRC cell lines of the CMS3 subtype incorporate ammonium into UMP at a higher rate than CMS2 CRC cell lines, consistent with observations of reduced expression of urea cycle enzymes in CRC [7]. Consequently, CMS3 organoids and CRC cell lines were more sensitive to pharmacological inhibition of CPS1, as well as inhibition of dihydroorotate dehydrogenase, another key enzyme of the pyrimidine biosynthesis pathway (Fig. 1). Hence, Torang and colleagues identified a subtype-specific metabolic dependency on de novo pyrimidine biosynthesis, adding to a growing body of evidence showing that CRC is amenable to interventions targeting key metabolic pathways with sensitivities sometimes restricted to specific genetic profiles [8–10].

Often, a good scientific work raises more questions than it answers. The paper by Torang and colleagues belongs to this category. For example, not all 16 GEMM genotypes were assigned to a subtype; some genotypes did not display associations with any specific subtype. This could be the outcome of the parameters used for specimen clustering, but also begs the question as to whether those GEMMs are unsuitable pre-clinical tools. Or perhaps, could those samples be related to human CRCs that are not classifiable within any CMS subtype? Moreover, Torang and colleagues showed that, even within the CMS3 group, mutations in *KRAS* lead to stronger expression of enterocyte gene signatures. It remains unclear what implications this has for the remaining *KRAS* wild-type specimens and whether sensitivity to CPS1 inhibition persists, as the authors only tested CRC cell lines carrying the mutant *KRAS* oncogene. The authors found a weak association between CMS3 and benefit from 5-fluorouracil (5-FU) based monotherapy. This observation ties in well with the highest reliance on de novo pyrimidine biosynthesis in this subtype and it warrants further investigation, as it might engender the identification of a subset of patients that would benefit from 5-FU-based adjuvant chemotherapy, as opposed to oxaliplatin-based interventions. Also, the nature of the intimate association between enterocytes and CMS3 cancer cells remains unclear. Do CMS3 cancers originate from the transformation of differentiated or precursor enterocytes, or are *KRAS* mutations conditioning and rewiring other cell types towards an enterocyte-like phenotype? And how does mutant *KRAS* affect the balance between the need for proliferation and the maintenance of a differentiated cell state? Interestingly, inhibition of the CPS1 enzyme in mouse organoids, together with impairing proliferation, increases the expression of enterocyte gene signatures. This would suggest a yet unappreciated association between the regulation of metabolic pathways and cell fate decisions. Finally, the observation that activation of Notch in a *KRAS*-mutant background drives a mesenchymal CMS4 subtype is of great interest and, as the authors themselves argue, warrants further examination of the role of Notch signalling in CRC and a potential crosstalk between CMS3 and CMS4 cancers [11].

Overall, Torang and colleagues’ paper has advanced our understanding of the properties and mechanisms underpinning CMS subtyping and their vulnerabilities. This will undoubtedly stimulate further fruitful research and prompt efforts towards the future development of subtype-specific treatments.

## Data Availability

No data were generated for the research described in this article.
